# Metagenomic Analysis of Silage

**DOI:** 10.3791/54936

**Published:** 2017-01-13

**Authors:** Richard K. Tennant, Christine M. Sambles, Georgina E. Diffey, Karen A. Moore, John Love

**Affiliations:** ^1^Biosciences, University of Exeter

**Keywords:** Genetics, Issue 119, metagenomics, DNA sequencing, shotgun sequencing, bioinformatics, silage, disease, livestock

## Abstract

Metagenomics is defined as the direct analysis of deoxyribonucleic acid (DNA) purified from environmental samples and enables taxonomic identification of the microbial communities present within them. Two main metagenomic approaches exist; sequencing the 16S rRNA gene coding region, which exhibits sufficient variation between taxa for identification, and shotgun sequencing, in which genomes of the organisms that are present in the sample are analyzed and ascribed to "operational taxonomic units"; species, genera or families depending on the extent of sequencing coverage.

In this study, shotgun sequencing was used to analyze the microbial community present in cattle silage and, coupled with a range of bioinformatics tools to quality check and filter the DNA sequence reads, perform taxonomic classification of the microbial populations present within the sampled silage, and achieve functional annotation of the sequences. These methods were employed to identify potentially harmful bacteria that existed within the silage, an indication of silage spoilage. If spoiled silage is not remediated, then upon ingestion it could be potentially fatal to the livestock.

**Figure:**
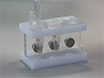


## Introduction

Metagenomics is the direct analysis of DNA purified from biological communities found within environmental samples ^1^ and was originally used to detect unculturable bacteria found in sediments ^2^. Metagenomics has been widely used for a number of applications, such as identifying the human microbiome ^3^, classifying microbial populations within the ocean ^4^ and even for the analysis of the bacterial communities that develop on coffee machines ^5^. The introduction of next generation sequencing technologies resulted in greater sequencing throughput and output. Consequently, DNA sequencing has become more economical ^6^ and the depth of sequencing that can be performed has greatly increased, enabling metagenomics to become a powerful, analytical tool.

"Front-end" enhancements in the practical, molecular aspect of metagenomic sequencing have driven the growth of the *in silico *bioinformatics tools available for the taxonomic classification ^7-9^, functional annotation ^10,11^ and visual representation ^12,13^ of DNA sequence data. The increasing number of available, sequenced prokaryotic and eukaryotic ^14^ genomes allows further accuracy in the classification of microbial communities, which are invariably performed against a "back-end" reference database of sequenced genomes ^15^. Two main approaches can be adopted for metagenomic analysis.

The more conventional method is analysis of the 16S rRNA gene coding region of bacterial genome. The 16S rRNA is highly conserved between prokaryote species but exhibits nine hyper-variable regions (V1 - V9) which can be exploited for species identification ^16^. The introduction of longer sequencing (≤ 300 bp paired end) allowed for the analysis of DNA sequences spanning two hyper-variable regions, in particular the V3 - V4 region ^17^. Advances in other sequencing technologies, such as Oxford Nanopore ^18^ and PacBIO ^19^, do allow the entire 16S rRNA gene to be sequenced contiguously.

While 16S rDNA based libraries provide a targeted approach to species identification and enable the detection of low copy number DNA that naturally occurs within purified samples, shotgun sequencing libraries allow for the detection of species that may contain DNA regions that are either not amplifiable by the 16S rRNA marker primer sequences used, or because the differences between the template sequence and the amplifying primer sequence are too great ^20,21^. Furthermore, although DNA polymerases have a high fidelity of DNA replication, base errors can nonetheless occur during PCR amplification and these incorporated errors can result in incorrect classification of originating species ^22^. Biases in the PCR amplification of template sequences can also occur; sequences of DNA with a high GC content can be under represented in the final amplicon pool ^23^ and similarly unnatural base modifications, such as thymine glycol, can halt DNA polymerases causing failures in the amplification of DNA sequences ^24^. In contrast, a shotgun sequencing DNA library is a DNA library that has been prepared by using all of the purified DNA that has been extracted from a sample and subsequently fragmented into shorter DNA chain lengths prior to preparation for sequencing. Taxonomic classification of DNA sequences generated by shotgun sequencing is more accurate when compared to 16S rRNA amplicon sequencing ^25^, although the financial cost required to reach a reliable sequencing depth is greater than that of amplicon sequencing ^26^. The major benefit of shotgun sequencing metagenomics is that sequenced regions of the various genomes in the sample are available for gene prospecting once they have been has been taxonomically classified ^27^.

Metagenomic sequence data is analyzed by an ever-increasing range of bioinformatic tools. These tools are able to perform a wide variety of applications, for example, quality control analysis of the raw sequence data ^28^, overlapping of paired end reads ^29^, *de novo* assembly of sequence reads to contigs and scaffolds ^30,31^, taxonomic classification and visualization of sequence reads and assembled sequences ^7,12,32,33^ and the functional annotation of assembled sequences ^34,35^.

Silage, produced by farmers throughout the world from fermented cereals such as maize (*Zea mays*), is predominately used as cattle feed. Silage is treated with the bacterium *Lactobacillus *sp. to aid fermentation ^36^ but to date, there is limited knowledge of the other microbial populations found in silage. The fermentation process can lead to undesirable and potentially harmful micro-organisms becoming prevalent within the silage ^37^. In addition to yeasts and molds, bacteria are particularly adaptable to the anaerobic environment in fermenting silage and are more frequently associated with diseases in livestock rather than the degradation of the silage ^38^. Butyric acid bacteria can be inadvertently added from soil remains when filling the silage silos and are able to convert the lactic acid, a product of anaerobic digestion, to butyric acid, thus increasing the pH of the silage^ 39^. This increase in pH can lead to an upsurge in spoilage bacteria that would normally be unable to sustain growth under optimum silage fermentation conditions ^38^. *Clostridium *spp*.*, *Listeria *spp*. *and *Bacillus *spp*.* are of particular concern, especially in silage for dairy cattle feed, as bacterial spores that have survived the gastrointestinal tract ^40^ can enter the food-chain, lead to food spoilage and, in rare cases, to animal and human fatalities ^37,39,41-44^. Moreover, while it is difficult to estimate the exact economic impact of veterinary treatment and livestock loss caused by silage spoilage, it is likely to be detrimental to a farm if an outbreak was to occur.

It is hypothesized that by using a metagenomic approach we can classify the microbial populations that are present in silage samples and furthermore identify microbial communities associated with silage spoilage that would, in turn, potentially have a detrimental effect on the livestock, enabling remedial action to be taken before the silage is to be used as a food source.

## Protocol

### 1. Site Location

1. Collect the silage sample from an appropriate site such as a farm. Here, the farm was located in Ballydulea, Co. Cork, Ireland (51°51'58.4"N 8°16'48.7"W).

### 2. DNA Extraction

NOTE: DNA extraction was performed using a commercial kit following the manufacturer's instructions. A negative control, which contained no sample, was used throughout the library preparation method.

1. Add 100 - 400 mg of sample to 978 µL sodium phosphate buffer and 122 µL soil lysis buffer in the supplied lysis tubes.
2. Homogenize samples by placing the lysis tubes into the homogenizer for 40 s at a speed of 6.0 m/s.
3. Centrifuge lysates at 14,000 x g for 15 min and transfer the supernatant to a clean micro-centrifuge tube containing 250 µL of Protein Precipitate Solution (PPS). Mix the solution by inverting 10 times and centrifuge at 14,000 x g for 5 min.
4. Add the supernatant to 1 mL DNA binding matrix in a clean 15 mL centrifuge tube. Mix the solution by inverting the tube constantly for 3 min. Allow the mixture to settle for 3 min, then discard 500 µL of supernatant. Mix the remaining supernatant.
5. Transfer 600 µL of the suspension to a spin filter and centrifuge at 14,000 x g for 1 min. Discard the filtrate and repeat the process with the remaining suspension.
6. Add 500 µL of wash buffer to the DNA binding matrix within the spin filter, mix by pipetting, then centrifuge at 14,000 x g for 1 min.
7. Discard the filtrate and centrifuge the spin filter again at 14,000 x g for 2 min to ensure all wash buffer is removed. Dry the spin filter at 23 ˚C for 5 min.
8. Pre-warm (70 ˚C) the DNase-free water (DES) and re-suspend the DNA binding matrix in 100 µL of DES within the spin filter. Transfer the spin filter to a clean 1.5 mL micro-centrifuge tube and centrifuge at 14,000 x g for 1 min to elute DNA. Store the purified DNA at -20 ˚C until further analysis is performed.

### 3. DNA Purification Using DNA Purification Beads

NOTE: Prior to metagenomic library preparation the extracted DNA was purified using purification beads to ensure a pure DNA sample was obtained.

1. Incubate the beads at 23 ˚C for 30 min before use. Add 2 volumes of beads to the DNA sample and incubate the solution at 23 ˚C for 5 min.
2. Place the samples onto a separation magnet for 5 min and then discard the supernatant. Wash the beads twice with 200 µL fresh 80% ethanol (EtOH). Air dry the beads for 10 min.
3. Remove the samples from the separation magnet and add 50 µL of elution buffer (EB), mix by pipetting.
4. Incubate the suspension at 23 ˚C for 5 min, after which place the samples back onto the separation magnet for 3 min.
5. Transfer the supernatant, which contains the DNA, to a clean tube. Discard the beads.
6. Quantify the purified DNA as per section four.

### 4. Quantification of Purified DNA

NOTE: Purified DNA was quantified using a fluorometer and double-stranded (dsDNA) High Sensitivity (HS) assay kit following the manufacturer's instructions.

1. Prepare a working solution using 199:1 ratio of buffer to reagent.
2. Add 10 µL of each DNA standard to 190 µL of working solution.
3. Add 10 µL of purified DNA to 190 µL of working solution. The final volume should be 200 µL. Incubate standard and DNA samples at 23 ˚C for 2 min.
4. Analyze standards before the DNA samples on the fluorometer using the on-screen instructions.

### 5. Shotgun Sequencing Library Preparation

NOTE: The shotgun sequencing library was prepared using a commercial library preparation kit using the manufacturer's instructions.

1. Dilute the DNA samples to 0.2 ng/µL using EB. Any sample which is already below this concentration, *i.e.* the negative control, is left at its current concentration.
2. Mix 5 µL of the purified DNA with 10 µL buffer and 5 µL enzyme mix. Incubate samples at 55 ˚C for 5 min.
3. Add 5 µL of neutralizing buffer and incubate the solution at 23 ˚C for 5 min.
4. Add 5 µL of each of the sample specific sequencing indices and 15 µL of PCR master mix.
5. In a thermocycler, incubate the samples at 72 ˚C for 3 min, 95 ˚C for 30 s, before 12 cycles of 95 ˚C for 10 s, 55 ˚C for 30 s and 72 ˚C for 30 s. Incubate samples finally at 72 ˚C for 5 min.
6. Purify the prepared DNA using the bead purification as before but with a final elution of 30 µL of EB.

### 6. Library Quantity and Quality Check

NOTE: The quantity and quality of the prepared libraries were assessed using a commercial kit and instrumentation.

1. Incubate the kit components at 23 ˚C for 30 min prior to use.
2. Add 2 µL of DNA to 2 µL of buffer and vortex for 1 min at 2,000 rpm.
3. Spin down the sample to ensure it is at the bottom of the tube.
4. Insert the sample tubes, analysis tape and tips into the instrument, and perform analysis as directed by the software.

### *7.* DNA Sequencing

1. Transfer the prepared and quantified DNA sequencing libraries samples to a sequencing service and sequence using 300 bp paired end sequencing ^45^.

### 8. Analysis of Raw Sequence Data

NOTE: The commands for each program using a Linux operating system are shown below the protocol step. The pipeline used for sequence data analysis is shown in **Figure 1**. The programs are to be installed by the user prior to analysis. This process should be performed individually for each sample.

1. Analyze and visualize DNA sequence data using FastQC ^46^ by typing in to the command line /path-to-file/fastqc, followed by the forward and reverse raw reads raw_read1.fastq raw_read2.fastq.
2. Specify an output folder by typing -o output_fastqc and the file format of the raw read files by -f fastq.
3. View the output file (**Figure 2**). path-to-file/fastqc raw_read1.fastq raw_read2.fastq -o output_directory -f fastq.

### 9. Quality Control Trimming and Filtering Sequence Data

1. Run the trimming program, Trimmomatic ^28^ by typing into the command line java -jar /path-to-file/ trimmomatic-0.35.jar.
2. Specify the files are paired end files by typing 'PE'. State that 16 central processing units (CPUs) should be used by the program by typing -threads 16.
3. List the two files to QC check by typing the names of the raw forward and reverse reads. The prefix of the output files is determined by typing -baseout silage.
4. Define the options for the program by typing ILLUMINACLIP:NexteraPE-PE.fa:2:30:10 LEADING:3 TRAILING:3 SLIDINGWINDOW:4:20 CROP:200 HEADCROP:15 MINLEN:36.
5. Once complete, analyze the trimmed sequences using FastQC as before and compare the output to the raw sequence data to ensure trimming has been performed successfully. NOTE: The software tool, Trimmomatic, trimmed reads further by removing leading low quality or N bases (below quality 3), removing trailing low quality or N bases (below quality 3) and scanning each read with a 4-base wide sliding window. The parameters were set for cutting when the average quality per base drops below 20 and then to drop any reads below 36 bases long. Finally, 15 bases were cropped from the head of each read and reads were cropped to keep 200 bases from the start of the read. This final step was performed to overcome some quality issues when sequencing long (> 200 bp) reads. These can be adjusted for specific samples ^28^. java -jar /path-to-file/trimmomatic-0.35.jar PE -threads 16 raw_read1.fastq raw_read2.fastq -baseout silage ILLUMINACLIP:NexteraPE-PE.fa:2:30:10 LEADING:3 TRAILING:3 SLIDINGWINDOW:4:20 CROP:200 HEADCROP:15 MINLEN:36

### 10. Metagenome Assembly

1. Merge the unpaired, trimmed reads by typing cat followed by the unpaired reads; silage_read1_unpaired.fastq silage_read2_unpaired.fastq. Write the files to a new file by typing > silage_merged_unpaired.fastq cat silage_read1_unpaired.fastq silage_read2_unpaired.fastq > silage_merged_unpaired.fastq
2. To *de novo* assemble the sequenced DNA, use SPAdes (St. Petersburg genome assembler) ^30^ by typing /path-to-file/spades.py. Specify that 16 CPUs are to be used by typing -t 16 and that the metagenomic parameter should applied by typing --meta.
3. Identify the trimmed forward reads using -1 silage_read1_paired.fastq and the reverse reads by -2 silage_read2_paired.fastq. The merged unpaired reads are specified by -s silage_merged_unpaired.fastq.
4. Define the output folder by typing -o silage_spades. path-to-file/spades.py -t 16 --meta -1 silage_read1_paired.fastq -2 silage_read2_paired.fastq -s silage_merged_unpaired.fastq -o silage_spades

### 11. Paired-end Read Overlap

1. Merge pairs of DNA sequence reads using FLASH (Fast Length Adjustment of Short Reads) ^29^ by typing into command line /path-to-file/flash. Specify that 16 CPUs should be used by using -t 16 and the output prefix by typing -o silage.
2. Identify trimmed reads by typing silage_trimmed_R1.fastq silage_trimmed_R2.fastq path-to-file/flash -t 16 -o FLASHed silage_read1_paired.fastq silage_read2_paired.fastq

### 12. Taxonomic Classification

1. Type /path-to-file/kraken and specify the database by typing --db /path-to-file/standard.
2. Define that 16 CPUs should be used by typing --threads 16 and identify an output folder by using --output FLASHed_silage_extendedFrags_kraken.txt. Type the input file name; FLASHed_silage.extendedFrags.fastq path-to-file/kraken --db standard --thread 16 --output FLASHed_silage_extendedFrags_kraken.txt FLASHed_silage.extendedFrags.fastq NOTE: Classification of the assembled DNA sequence scaffolds using Kraken ^7^ was completed against the most recent, standard Kraken database that contained all available *Prokaryote *genome sequences.
3. Transfer columns 2 and 3 from the output file and to a new file by typing cut -f2,3 FLASHed_silage_extendedFrags_kraken.txt > FLASHed_silage_extendedFrags_kraken.int
4. Open the output file in web browser. cut -f2,3 FLASHed_silage_extendedFrags_kraken.txt > FLASHed_silage_extendedFrags_kraken.int
5. Import the new file into Krona ^12^ by typing ktImportTaxonomy. Specify the input file by typing FLASHed_silage_extendedFrags_kraken.int. Identify the output file by typing -o FLASHed_silage_extendedFrags_kraken.out.html. path-to-file/ktImportTaxonomy FLASHed_silage_extendedFrags_kraken.int -o FLASHed_silage_extendedFrags_kraken.out.html

### 13. Functional Annotation

1. Go to the MG-RAST ^47^ website, http://metagenomics.anl.gov/. Register as a new user if required. After logging in, Click on the “Upload” button. Upload the assembled scaffolds from Step 10.
2. Once the files have uploaded, click on "Submit" and follow the instructions and await the completion of analysis.
3. After the analysis is complete, view the link sent *via* email from MG-RAST, or alternatively, click on "Progress". There is a list of completed jobs. Click on the relevant job id and then on the link to the "download page".
4. On the download page, under the heading "Protein Clustering 90%", click on the protein button to download the predicted protein file, 550.cluster.aa90.faa.
5. To classify the proteins as putatively belonging to a particular CAZy enzyme class, compare the downloaded proteins to the CAZy database ^48^. Download the Carbohydrate-Active enZYmes Database (CAZy) from files are: AA.zip, CE.zip, GH.zip, GT.zip and PL.zip. These files represent the following enzyme classes respectively: Auxiliary Activities (AA), Carbohydrate Esterases (CE), Glycoside Hydrolases (GH), Glycosyl Transferases (GT) and Polysaccharide Lyases (PL).
6. Unzip the database files and annotate the proteins by determining the protein similarity to the CAZy database proteins using the USEARCH UBLAST algorithm ^49^. To use a bash loop (for i in *.txt) to iterate through the 5 database .txt files type "for i in *.txt; do".
7. Run USEARCH by typing /path-to-file/usearch8 with the parameter -ublast in order to use the ublast algorithm. Then type in the name of the protein sequence file downloaded from MG-RAST, "mgmXXXXXX.3.550.cluster.aa90.faa".
8. To indicate the database file to be used type "-db $i" and to specify the E-value threshold at 1e^-5^, type "-evalue 1e-5".
9. To terminate the search after the discovery of a target sequence and therefore classifying that protein sequence as belonging to the target enzyme class, *e.g.* GH, type "-masaccepts 1".
10. To define that 16 CPUs should be used type "-threads 16" and to specify the format of the output file as atab-separated text type "-blast6out". To identify the output file type "$i.ublast". To terminate the bash loop, type "; done" for i in *.txt; do /path-to-file/usearch8 -ublast ../mgmXXXXXX.3.550.cluster.aa90.faa -db $i -evalue 1e-5 -maxaccepts 1 -threads 16 -blast6out $i.ublast; done

### 14. Visualizing CAZy Annotation

1. To visualize the output from the CAZy annotation as a Venn diagram, generate protein ID lists for each enzyme class using a bash loop. Type "for i in *.ublast; do".
2. To transfer column 1 from the output file and to a new file, type "cat $i | cut -f 1 >$i.list".
3. Terminate the loop and type "; done".
4. Open the .list files in a text editor. Go to the website , select the number of sets as 5 and paste the content of each list file in a separate box. Download the resulting diagram as a .SVG file. for i in *.ublast; do cat $i | cut -f 1 >$i.list; done

## Representative Results

Prior to bioinformatic processing, raw sequence reads were trimmed and adapters were removed using Trimmomatic software ^28^. After the trimming and filtering step, the number of reads was reduced to 50% of the sequence reads (**Table 1**). The average base phred score was >30 after quality control (**Figure 2**).

Pairs of DNA sequences which had overlapping regions were merged using FLASH software ^29^ to generate single longer reads, non-overlapping reads were kept in a separate file. 45.47% reads (105,343) combined successfully. Following the overlapping of reads using FLASH of reads, the resulting extended fragments underwent bacterial taxonomic classification using Kraken software ^7^ and were subsequently visualized with Krona software (**Figure 3**).

The majority of the bacterial species present in the silage metagenome are found within 4 prokaryotic phyla: Firmicutes (34%), Actinobacteria (28%), Proteobacteria (27%) and Bacteroidetes (7%). The distribution of classes present within these phyla can be seen in **Figure 4**. The most abundant species in the metagenome were *Lactobacillus* spp. (24%; Firmicutes), *Corynebacterium* spp. (8%; Actinobacteria), *Propionibacterium* spp. (3%; Actinobacteria) and *Prevotella* spp. (3%; Bacteroidetes). Species important to animal health and implicated in disease were also observed; *Clostridium* spp. (1%) *Bacillus* spp. (0.6%), *Listeria* spp. (0.2%) were predicted to be present in the silage sample.

Functional annotation was performed on assembled reads. The metagenome was assembled using the SPAdes assembler ^30^ using the trimmed and filtered paired-end and unpaired reads generating 92,284 scaffolds. In order to identify cellulases, proteins were predicted using MG-RAST and annotated using the Carbohydrate-Active enZYmes Database (CAZy). Of the 97,562 predicted proteins, 6357 were annotated as a putative carbohydrate-active enzyme in one of the five enzymes classes that make up the CAZy database (**Figure 5**). Results were visualized as a Venn diagram using InteractiVenn software ^50^ showing the distribution of protein annotations including those containing more than one CAZy enzyme class annotation. Of these, 3861 were predicted to have glycoside hydrolase activity and will be further characterized in the laboratory to confirm function.


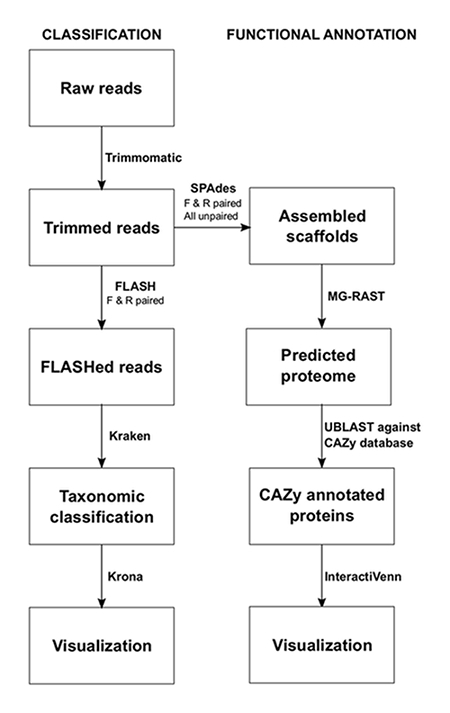
**Figure 1:Bioinformatic Metagenomics Pipeline for the Analysis of Silage. **Two main approaches were used to investigate the microbiome of silage, taxonomic classification and functional annotation. Please click here to view a larger version of this figure.


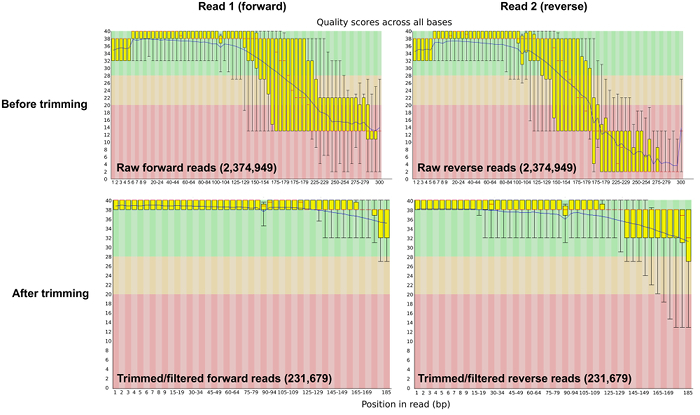
**Figure 2:Sequence Quality Per-base Before and After Trimming and Adapter Removal. **The per-base sequence quality plot from FASTQC shows the average phred score across the length of the sequence reads pre- and post- quality control. Please click here to view a larger version of this figure.


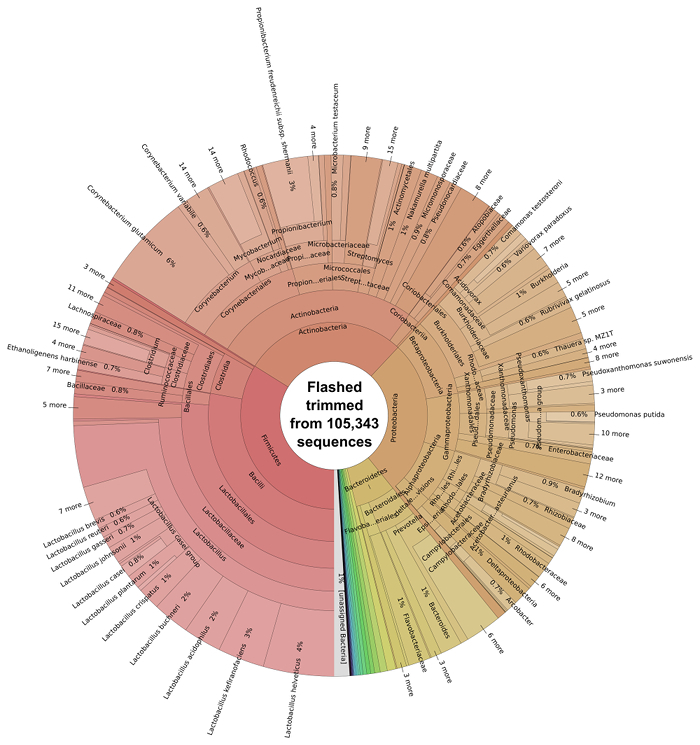
**Figure 3:Taxonomic Classification of the Bacterial Microbiome of Solid Silage. **Classification of trimmed and overlapping sequence reads from FLASH was performed using Kraken ^7^ and subsequently visualized with krona. Please click here to view a larger version of this figure.


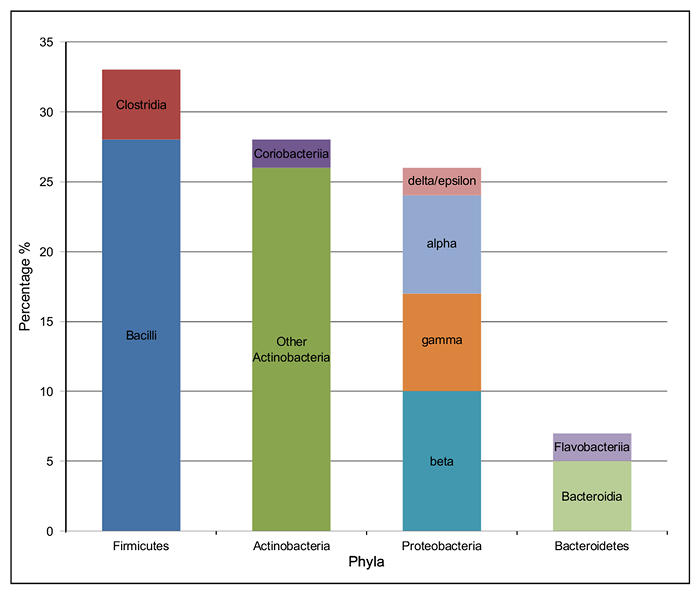
**Figure 4:Taxonomic Class Distribution of the 4 Most Abundant Phyla in the Bacterial Microbiome of Solid Silage. **The percentage of each class of bacteria within the four most abundant phyla. Firmicutes: *Clostridia *(red) and *Bacilli* (dark blue); Proteobacteria: *delta/epsilon* (pink), *alpha* (pale blue), *gamma* (orange) and *beta *(turquoise); Bacteroidetes: *Flavobacteriia* (dark blue) and *Bacteroidia* (pale green); Actinobacteria: *Coriobacteriia* (dark purple) and other *Actinobacteria* (dark green). Please click here to view a larger version of this figure.


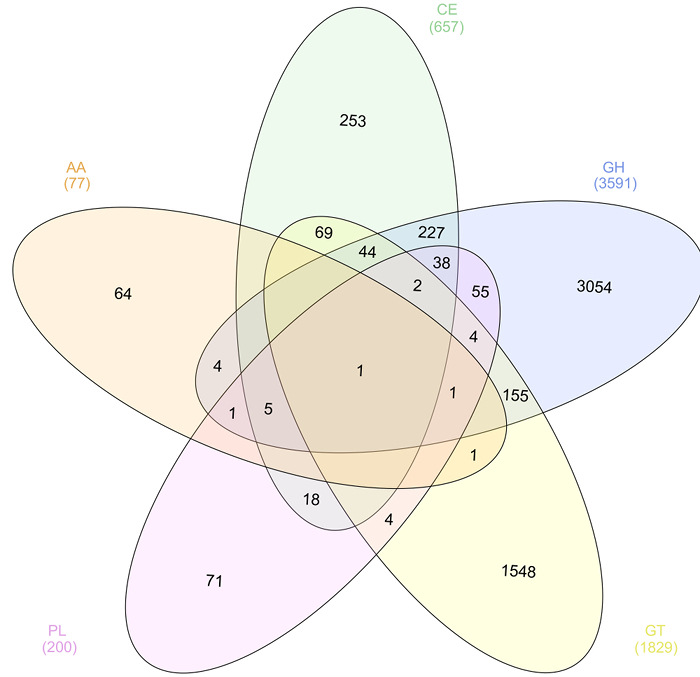
**Figure 5:CAZy Annotation of the Predicted Proteome in the Solid Silage Microbiome. **Venn diagram showing the distribution of the five enzyme classes of CAZy annotations in the predicted proteome of solid silage microbiome. Please click here to view a larger version of this figure.

**Table:** 

# Raw reads	# Filtered reads (paired)	# Filtered reads	# FLASHed reads
(paired)	(unpaired)
2,374,949 x2	231,679 x2	1,892,534	105,343

**Table 1: Summary Table of Sequencing Reads.**

## Discussion

While an *in silico* analysis can give an excellent insight to the microbial communities that are present within environmental samples, it is critical that the taxonomic classifications demonstrated be performed in association with relevant controls and that a suitable depth of sequencing has been achieved to capture the entire population present ^51^.

With any computational analysis, there are many routes to achieve a similar goal. The methods that we have used in this study are *examples* of suitable and straightforward methods, that have been brought together to achieve a range of analyses on the silage microbiome. A variety and an ever-increasing number of bioinformatics tools and techniques are available to analyze metagenomic data, for instance Phylosift ^8^ and MetaPhlAn2 ^52^, and these should be evaluated prior to the investigation for their relevance to the sample and the analysis required ^53^. Metagenomic analysis methods are limited by the databases for available for classification, sequencing depth and the quality of sequencing.

The bioinformatic processing demonstrated here was performed on a local, high powered machine; however cloud-based systems are also available. These cloud-based services allow for the rental of the necessary computational power without having the high-cost investment of a suitable powerful local workstation. A potential application of this method would be to assess silage before its use in agriculture to ensure that no potentially harmful bacteria are present therefore preventing them from entering the food chain.

## Disclosures

The authors have nothing to disclose.
